# 1-(8-Bromo-2-methyl-4-thioxo-3,4,5,6-tetra­hydro-2*H*-2,6-methano-1,3-benzoxazocin-11-yl)ethanone

**DOI:** 10.1107/S160053680900347X

**Published:** 2009-02-04

**Authors:** G. V. Palamarchuk, O. V. Borisov, S. S. Kovalenko, V. P. Chernykh, S. M. Kovalenko, V. N. Baumer, O. V. Shishkin

**Affiliations:** a STC Institute for Single Crystals, National Academy of Sciences of Ukraine, 60 Lenina Avenue, Kharkiv 61001, Ukraine; b Department of Pharmaceutical Chemistry, National University of Pharmacy, 4 Blyukhera Avenue, Kharkiv 61002, Ukraine

## Abstract

In the title compound, C_14_H_14_BrNO_2_S, there are two similar non-equivalent mol­ecules in the asymmetric unit, displaying three chiral centres each. In the crystal structure, they are linked by inter­molecular N—H⋯O hydrogen bonds to form infinite chains, which are in turn connected by weak Br⋯H and S⋯H inter­actions.

## Related literature

For related literature on the applications of thio­phene derivatives, see: Zaragoza Dorwald (2000 ▶); Kovalenko & Victorova (2005 ▶). For analogous conformations, see: Bilokin *et al.* (1988 ▶); Raev *et al.* (2004 ▶); Biala *et al.* (2002 ▶); Konovalova *et al.* (2007 ▶); O’Callaghan *et al.* (1997 ▶); Zefirov & Zorky (1995 ▶).
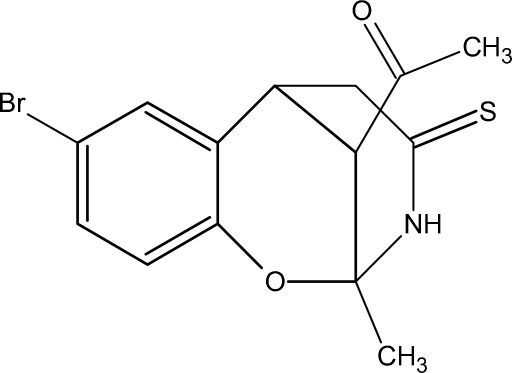


## Experimental

### 

#### Crystal data

C_14_H_14_BrNO_2_S
*M* *_r_* = 340.23
Triclinic, 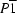

*a* = 8.213 (5) Å
*b* = 11.625 (7) Å
*c* = 15.156 (10) Å
α = 98.67 (5)°
β = 99.09 (5)°
γ = 101.81 (5)°
*V* = 1373.1 (15) Å^3^
*Z* = 4
Mo *K*α radiation
μ = 3.14 mm^−1^
*T* = 293 (2) K
0.6 × 0.1 × 0.05 mm

#### Data collection

Siemens *P*3/PC diffractometer
Absorption correction: integration (*XPREP*; Siemens, 1991 ▶) *T*_min_ = 0.611, *T*_max_ = 0.855
7869 measured reflections
4773 independent reflections
3383 reflections with *I* > 2/s(*I*)
*R*_int_ = 0.012
2 standard reflections every 98 reflections intensity decay: 1%

#### Refinement

*R*[*F* ^2^ > 2σ(*F* ^2^)] = 0.052
*wR*(*F* ^2^) = 0.118
*S* = 1.03
4773 reflections
348 parameters
H-atom parameters constrained
Δρ_max_ = 0.37 e Å^−3^
Δρ_min_ = −0.43 e Å^−3^

### 

Data collection: *P3* (Siemens, 1991 ▶); cell refinement: *P3*; data reduction: *XDISK* and *XPREP* (Siemens, 1991 ▶); program(s) used to solve structure: *SHELXS97* (Sheldrick, 2008 ▶); program(s) used to refine structure: *SHELXL97* (Sheldrick, 2008 ▶); molecular graphics: *XP* in *SHELXTL* (Sheldrick, 2008 ▶); software used to prepare material for publication: *SHELXL97*.

## Supplementary Material

Crystal structure: contains datablocks I, global. DOI: 10.1107/S160053680900347X/bg2221sup1.cif

Structure factors: contains datablocks I. DOI: 10.1107/S160053680900347X/bg2221Isup2.hkl

Additional supplementary materials:  crystallographic information; 3D view; checkCIF report

## Figures and Tables

**Table 1:** Hydrogen-bond geometry (Å, °)

*D*—H⋯*A*	*D*—H	H⋯*A*	*D*⋯*A*	*D*—H⋯*A*
N10*A*—H10*A*⋯O14*B*^i^	0.86	2.20	2.981 (4)	150
N10*B*—H10*B*⋯O14*A*^ii^	0.86	2.15	2.960 (4)	157

Symmetry codes: (i) 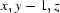; (ii) 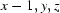.

**Fig. 1.**
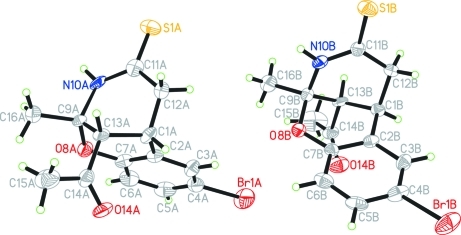
View of the title compound with atomic numbering. Displacement ellipsoids drawn at a 50% probability level.

## supplementary crystallographic information

### Comment

The fragment of thiophene is a very important pharmacophore part of many biologically active compounds. Thiophene derivatives are used as antitussives (Zaragoza Dorwald, 2000; Kovalenko & Victorova, 2005), antibiotics, anaesthetics, antiparasitics, resolvents, anthelmintic drugs, anticholinergic drugs, antiulcer agents, antihistamines. Investigation of the molecular structure of these compounds may provide useful information for understanding the mechanism of their biological activity. In this paper we report the molecular and crystal structure of the 2-aroyl-3-amino-4-arylsulfonyl-5-arylamino-thiophene. There are two molecules in the asymmetric unit (labelled A and B in Fig. 1), with a similar distribution of chiral centers (C1, C9 and C13). Both molecules have similar geometrical characteristics: the piperidine-2-tione and tetrahydropyran rings adopt a half-chair conformation; deviations of the C1 and C13 atoms of the tetrahydropyrimidine ring from the mean-square planes of the remaining atoms in the ring are -0.46 (1) Å, 0.44 (1) Å and 0.37 (1) Å, -0.51 (1) Å in molecules A and B, respectively. Deviations of the C9 and C13 atoms of the tetrahydropyrane from the mean-square planes of the remaining atoms in the rings are -0.36 (1) Å, 0.44 (1) Å, in both molecules. The two rings are fused in such way that the C16 methyl group and the H atom at the C1 have equatorial orientation (the C7—C8—C9—C16 and C7—C2—C1—H1A torsion angles being -177.2 (3)° and -140.7 (3)° (molecule A) and -175.2 (3)° and -140.0 (3)° (molecule B). The same type of ring fusion have been observed in related compounds (Konovalova *et al.*, 2007; Raev *et al.*, 2004; Bilokin *et al.*, 1988; O'Callaghan *et al.*, 1997; Biala *et al.*, 2002). The C13—C14 bond has an equatorial orientation with regard to the piperidine-2-tione ring (the N10—C9—C13—C14 torsion angle being -176.6 (3)° and -179.8 (3)° (A, B respectively). The acetyl group adopts an orthogonal arrangement relative to the C9—C13 bond (the C9—C13—C14—O14 torsion angle being 92.4 (4)°, -89.8 (4)° (A, B respectively). The main H-bonding interactions are presented in Table 1. Molecules pack as infinite chains of alternating A and B molecules, due to intrachain N—H···O and weak C—H···Br interactions. Neighbouring chains in turn are connected by weak C—H···.S interactions as well as by stacking interactions between phenyl rings with an interplanar distance of 3.37 (1) Å.

### Experimental

The title compound was obtained by one-pot synthesis, starting from a mixture of 1 mmol 6-bromocoumarine-3-thioamide and 1.2 mmol of 2,4-pentanedione in 5 ml of methanol containing catalytic amounts of piperidine, which was refluxed for 5 min. Then it was cooled to 500 C and 2 mmol of potassium alkali was added. The reaction mixture was stirred at 500 C for 6 h (monitored by TLC). Then it was cooled to r.t. and diluted with water. Formed precipitate was filtered and washed with water and water–methanol, 1:1.

### Refinement

All H atoms were located from an electron density difference map and included in the refinement in the riding motion approximation with *U*_iso_ constrained to be 1.5 times *U*_eq_ of the carrier atom for the methyl groups and 1.2 times *U*_eq_ of the carrier atom for the other atoms.

### Crystal data

**Table:**

C_14_H_14_BrNO_2_S	*Z* = 4
*M**_r_* = 340.23	*F*(000) = 688
Triclinic, *P*1	*D*_x_ = 1.646 Mg m^−^^3^
Hall symbol: -P 1	Mo *K*α radiation, λ = 0.71073 Å
*a* = 8.213 (5) Å	Cell parameters from 24 reflections
*b* = 11.625 (7) Å	θ = 10–11°
*c* = 15.156 (10) Å	µ = 3.14 mm^−^^1^
α = 98.67 (5)°	*T* = 293 K
β = 99.09 (5)°	Needle, colourless
γ = 101.81 (5)°	0.6 × 0.1 × 0.05 mm
*V* = 1373.1 (15) Å^3^	

### Data collection

**Table:**

Siemens P3/PC diffractometer	3383 reflections with *I* > 2/s(*I*)
Radiation source: sealed tube	*R*_int_ = 0.012
graphite	θ_max_ = 25.0°, θ_min_ = 2.1°
2θ/θ scans	*h* = −9→4
Absorption correction: integration (*XPREP*; Siemens, 1991)	*k* = −13→13
*T*_min_ = 0.611, *T*_max_ = 0.855	*l* = −18→18
7869 measured reflections	2 standard reflections every 98 reflections
4773 independent reflections	intensity decay: 1%

### Refinement

**Table:**

Refinement on *F*^2^	Secondary atom site location: difference Fourier map
Least-squares matrix: full	Hydrogen site location: inferred from neighbouring sites
*R*[*F*^2^ > 2σ(*F*^2^)] = 0.052	H-atom parameters constrained
*wR*(*F*^2^) = 0.118	*w* = 1/[σ^2^(*F*_o_^2^) + (0.0597*P*)^2^ + 0.9457*P*] where *P* = (*F*_o_^2^ + 2*F*_c_^2^)/3
*S* = 1.03	(Δ/σ)_max_ = 0.001
4773 reflections	Δρ_max_ = 0.37 e Å^−^^3^
348 parameters	Δρ_min_ = −0.43 e Å^−^^3^
0 restraints	Extinction correction: *SHELXL97* (Sheldrick, 2008), Fc^*^=kFc[1+0.001xFc^2^λ^3^/sin(2θ)]^-1/4^
Primary atom site location: structure-invariant direct methods	Extinction coefficient: 0.0026 (5)

### Special details

**Table:**

Geometry. All e.s.d.'s (except the e.s.d. in the dihedral angle between two l.s. planes) are estimated using the full covariance matrix. The cell e.s.d.'s are taken into account individually in the estimation of e.s.d.'s in distances, angles and torsion angles; correlations between e.s.d.'s in cell parameters are only used when they are defined by crystal symmetry. An approximate (isotropic) treatment of cell e.s.d.'s is used for estimating e.s.d.'s involving l.s. planes.
Refinement. Refinement of *F*^2^ against ALL reflections. The weighted *R*-factor *wR* and goodness of fit *S* are based on *F*^2^, conventional *R*-factors *R* are based on *F*, with *F* set to zero for negative *F*^2^. The threshold expression of *F*^2^ > σ(*F*^2^) is used only for calculating *R*-factors(gt) *etc*. and is not relevant to the choice of reflections for refinement. *R*-factors based on *F*^2^ are statistically about twice as large as those based on *F*, and *R*- factors based on ALL data will be even larger.

### Fractional atomic coordinates and isotropic or equivalent isotropic displacement parameters (Å^2^)

**Table:**

	*x*	*y*	*z*	*U*_iso_*/*U*_eq_	
Br1A	0.62044 (6)	0.83270 (4)	0.58068 (4)	0.05907 (14)	
S1A	0.17210 (13)	0.30146 (10)	0.67922 (7)	0.0413 (3)	
C1A	0.6441 (5)	0.5017 (3)	0.7900 (2)	0.0269 (8)	
H1A	0.6925	0.5695	0.8406	0.032*	
C2A	0.6686 (4)	0.5407 (3)	0.7023 (2)	0.0273 (8)	
C3A	0.6433 (5)	0.6509 (3)	0.6851 (2)	0.0295 (9)	
H3A	0.6122	0.7024	0.7291	0.035*	
C4A	0.6641 (5)	0.6833 (3)	0.6045 (3)	0.0346 (9)	
C5A	0.7090 (5)	0.6098 (4)	0.5373 (3)	0.0421 (11)	
H5A	0.7218	0.6340	0.4825	0.050*	
C6A	0.7350 (5)	0.4992 (4)	0.5518 (2)	0.0373 (10)	
H6A	0.7678	0.4488	0.5077	0.045*	
C7A	0.7109 (4)	0.4659 (3)	0.6333 (2)	0.0271 (8)	
O8A	0.7374 (3)	0.3533 (2)	0.64432 (16)	0.0299 (6)	
C9A	0.6713 (4)	0.3045 (3)	0.7161 (2)	0.0259 (8)	
N10A	0.4847 (4)	0.2771 (2)	0.69181 (19)	0.0284 (7)	
H10A	0.4371	0.2105	0.6551	0.034*	
C11A	0.3799 (5)	0.3432 (3)	0.7199 (2)	0.0302 (9)	
C12A	0.4574 (5)	0.4563 (3)	0.7894 (2)	0.0315 (9)	
H12A	0.4436	0.4411	0.8493	0.038*	
H12B	0.3979	0.5175	0.7762	0.038*	
C13A	0.7290 (4)	0.3964 (3)	0.8053 (2)	0.0264 (8)	
H13A	0.6831	0.3599	0.8531	0.032*	
C14A	0.9203 (5)	0.4390 (3)	0.8362 (2)	0.0307 (9)	
O14A	1.0003 (3)	0.5252 (2)	0.8167 (2)	0.0447 (8)	
C15A	1.0060 (6)	0.3680 (4)	0.8959 (3)	0.0531 (13)	
H15A	1.1254	0.4039	0.9121	0.080*	
H15B	0.9881	0.2874	0.8637	0.080*	
H15C	0.9592	0.3675	0.9501	0.080*	
C16A	0.7249 (5)	0.1878 (3)	0.7156 (3)	0.0370 (10)	
H16A	0.6847	0.1382	0.6564	0.056*	
H16B	0.6777	0.1476	0.7599	0.056*	
H16C	0.8464	0.2035	0.7304	0.056*	
Br1B	0.59308 (7)	1.32613 (4)	1.02809 (3)	0.05332 (14)	
S1B	−0.10276 (13)	0.80780 (9)	0.93052 (6)	0.0360 (2)	
C1B	0.1321 (4)	1.0087 (3)	0.7679 (2)	0.0230 (8)	
H1AA	0.1125	1.0779	0.7416	0.028*	
C2B	0.3048 (4)	1.0401 (3)	0.8293 (2)	0.0252 (8)	
C3B	0.3636 (5)	1.1495 (3)	0.8877 (2)	0.0278 (9)	
H3AA	0.2963	1.2048	0.8891	0.033*	
C4B	0.5208 (5)	1.1774 (3)	0.9439 (2)	0.0356 (10)	
C5B	0.6249 (4)	1.0974 (3)	0.9440 (2)	0.0320 (9)	
H5AA	0.7312	1.1168	0.9820	0.038*	
C6B	0.5661 (5)	0.9884 (3)	0.8862 (2)	0.0319 (9)	
H6AA	0.6343	0.9337	0.8845	0.038*	
C7B	0.4067 (4)	0.9585 (3)	0.8304 (2)	0.0224 (8)	
O8B	0.3583 (3)	0.84740 (19)	0.77593 (16)	0.0274 (6)	
C9B	0.1825 (4)	0.8068 (3)	0.7368 (2)	0.0271 (8)	
N10B	0.0844 (4)	0.7827 (2)	0.8077 (2)	0.0293 (7)	
H10B	0.0821	0.7148	0.8240	0.035*	
C11B	−0.0017 (4)	0.8516 (3)	0.8502 (2)	0.0266 (8)	
C12B	−0.0047 (4)	0.9703 (3)	0.8219 (2)	0.0271 (8)	
H12C	0.0108	1.0312	0.8759	0.032*	
H12D	−0.1150	0.9649	0.7852	0.032*	
C13B	0.1177 (4)	0.9023 (3)	0.6920 (2)	0.0235 (8)	
H13B	−0.0029	0.8700	0.6653	0.028*	
C14B	0.2102 (4)	0.9365 (3)	0.6148 (2)	0.0291 (9)	
O14B	0.3348 (4)	1.0153 (2)	0.63118 (17)	0.0438 (8)	
C15B	0.1367 (7)	0.8714 (5)	0.5217 (3)	0.0717 (17)	
H15D	0.1654	0.9222	0.4793	0.108*	
H15F	0.0156	0.8478	0.5145	0.108*	
H15G	0.1811	0.8015	0.5102	0.108*	
C16B	0.1689 (5)	0.6886 (3)	0.6746 (3)	0.0413 (11)	
H16F	0.2055	0.6334	0.7100	0.062*	
H16G	0.2394	0.7013	0.6304	0.062*	
H16D	0.0532	0.6563	0.6439	0.062*	

### Atomic displacement parameters (Å^2^)

**Table:**

	*U*^11^	*U*^22^	*U*^33^	*U*^12^	*U*^13^	*U*^23^
Br1A	0.0566 (3)	0.0473 (2)	0.0811 (3)	0.0145 (2)	0.0039 (2)	0.0429 (2)
S1A	0.0277 (5)	0.0528 (6)	0.0445 (5)	0.0050 (4)	0.0101 (4)	0.0157 (5)
C1A	0.038 (2)	0.0186 (15)	0.0213 (16)	0.0018 (14)	0.0007 (15)	0.0060 (13)
C2A	0.0252 (19)	0.0281 (17)	0.0269 (17)	0.0003 (14)	0.0060 (15)	0.0076 (14)
C3A	0.035 (2)	0.0262 (17)	0.0256 (17)	0.0073 (15)	−0.0003 (15)	0.0065 (14)
C4A	0.0252 (19)	0.0378 (19)	0.044 (2)	0.0079 (16)	0.0015 (16)	0.0211 (16)
C5A	0.036 (2)	0.056 (2)	0.0323 (19)	−0.0007 (19)	0.0027 (17)	0.0205 (18)
C6A	0.039 (2)	0.048 (2)	0.0257 (17)	0.0051 (18)	0.0129 (16)	0.0090 (16)
C7A	0.0290 (19)	0.0293 (17)	0.0186 (15)	−0.0024 (15)	0.0033 (14)	0.0046 (13)
O8A	0.0365 (14)	0.0249 (12)	0.0318 (12)	0.0068 (10)	0.0166 (11)	0.0064 (10)
C9A	0.0244 (18)	0.0211 (15)	0.0372 (18)	0.0032 (14)	0.0144 (15)	0.0151 (13)
N10A	0.0354 (17)	0.0108 (13)	0.0313 (15)	−0.0076 (12)	0.0090 (13)	−0.0047 (11)
C11A	0.046 (2)	0.0345 (18)	0.0158 (15)	0.0102 (17)	0.0150 (15)	0.0116 (13)
C12A	0.033 (2)	0.0260 (17)	0.0356 (19)	0.0086 (15)	0.0080 (16)	0.0041 (15)
C13A	0.0279 (19)	0.0251 (16)	0.0298 (17)	0.0018 (14)	0.0173 (15)	0.0107 (13)
C14A	0.036 (2)	0.0220 (17)	0.0316 (18)	0.0062 (15)	0.0043 (16)	−0.0001 (14)
O14A	0.0347 (15)	0.0250 (13)	0.0719 (19)	−0.0046 (11)	0.0084 (14)	0.0193 (13)
C15A	0.044 (3)	0.055 (2)	0.061 (3)	0.001 (2)	0.003 (2)	0.033 (2)
C16A	0.036 (2)	0.0302 (19)	0.050 (2)	0.0109 (17)	0.0164 (18)	0.0104 (16)
Br1B	0.0680 (3)	0.0420 (2)	0.0324 (2)	−0.0129 (2)	0.0054 (2)	−0.00752 (17)
S1B	0.0361 (5)	0.0435 (5)	0.0338 (5)	0.0083 (4)	0.0157 (4)	0.0162 (4)
C1B	0.0162 (16)	0.0259 (16)	0.0266 (17)	0.0057 (13)	−0.0005 (14)	0.0078 (13)
C2B	0.0258 (18)	0.0229 (16)	0.0275 (17)	0.0009 (14)	0.0081 (14)	0.0094 (13)
C3B	0.035 (2)	0.0235 (16)	0.0233 (17)	0.0039 (15)	0.0067 (15)	0.0017 (14)
C4B	0.045 (2)	0.036 (2)	0.0156 (16)	−0.0109 (18)	0.0105 (16)	−0.0029 (14)
C5B	0.0175 (18)	0.049 (2)	0.0271 (18)	0.0001 (16)	−0.0012 (15)	0.0148 (16)
C6B	0.0256 (19)	0.0402 (19)	0.0355 (18)	0.0083 (16)	0.0083 (15)	0.0213 (15)
C7B	0.0206 (17)	0.0233 (16)	0.0252 (16)	0.0034 (13)	0.0091 (14)	0.0083 (13)
O8B	0.0236 (12)	0.0231 (11)	0.0378 (13)	0.0065 (10)	0.0077 (10)	0.0098 (10)
C9B	0.0232 (18)	0.0199 (16)	0.0378 (19)	0.0051 (14)	0.0061 (15)	0.0039 (14)
N10B	0.0295 (16)	0.0202 (13)	0.0422 (16)	0.0036 (12)	0.0123 (13)	0.0152 (12)
C11B	0.0239 (18)	0.0236 (16)	0.0357 (18)	0.0079 (14)	0.0061 (15)	0.0123 (14)
C12B	0.0209 (18)	0.0268 (17)	0.0348 (18)	0.0041 (14)	0.0104 (15)	0.0067 (14)
C13B	0.0187 (17)	0.0192 (15)	0.0307 (17)	−0.0035 (13)	0.0035 (14)	0.0106 (13)
C14B	0.0251 (19)	0.0276 (17)	0.0364 (19)	0.0035 (15)	0.0041 (15)	0.0175 (14)
O14B	0.0454 (17)	0.0466 (16)	0.0330 (14)	−0.0042 (14)	0.0131 (13)	0.0027 (12)
C15B	0.071 (4)	0.100 (4)	0.031 (2)	−0.007 (3)	0.010 (2)	0.007 (3)
C16B	0.041 (2)	0.034 (2)	0.047 (2)	0.0061 (18)	0.0116 (19)	0.0016 (18)

### Geometric parameters (Å, °)

**Table:**

Br1A—C4A	1.918 (4)	Br1B—C4B	1.909 (4)
S1A—C11A	1.663 (4)	S1B—C11B	1.662 (4)
C1A—C2A	1.497 (5)	C1B—C2B	1.509 (5)
C1A—C12A	1.514 (5)	C1B—C12B	1.525 (5)
C1A—C13A	1.556 (5)	C1B—C13B	1.528 (5)
C1A—H1A	0.9800	C1B—H1AA	0.9800
C2A—C7A	1.390 (5)	C2B—C3B	1.381 (5)
C2A—C3A	1.394 (5)	C2B—C7B	1.388 (5)
C3A—C4A	1.357 (5)	C3B—C4B	1.377 (5)
C3A—H3A	0.9300	C3B—H3AA	0.9300
C4A—C5A	1.374 (6)	C4B—C5B	1.387 (6)
C5A—C6A	1.390 (6)	C5B—C6B	1.373 (5)
C5A—H5A	0.9300	C5B—H5AA	0.9300
C6A—C7A	1.380 (5)	C6B—C7B	1.387 (5)
C6A—H6A	0.9300	C6B—H6AA	0.9300
C7A—O8A	1.400 (4)	C7B—O8B	1.368 (4)
O8A—C9A	1.431 (4)	O8B—C9B	1.423 (4)
C9A—N10A	1.474 (4)	C9B—N10B	1.468 (5)
C9A—C16A	1.509 (5)	C9B—C16B	1.518 (5)
C9A—C13A	1.532 (5)	C9B—C13B	1.526 (5)
N10A—C11A	1.342 (5)	N10B—C11B	1.332 (5)
N10A—H10A	0.8600	N10B—H10B	0.8600
C11A—C12A	1.507 (5)	C11B—C12B	1.510 (5)
C12A—H12A	0.9700	C12B—H12C	0.9700
C12A—H12B	0.9700	C12B—H12D	0.9700
C13A—C14A	1.521 (5)	C13B—C14B	1.549 (5)
C13A—H13A	0.9800	C13B—H13B	0.9800
C14A—O14A	1.186 (4)	C14B—O14B	1.190 (4)
C14A—C15A	1.501 (6)	C14B—C15B	1.467 (6)
C15A—H15A	0.9600	C15B—H15D	0.9600
C15A—H15B	0.9600	C15B—H15F	0.9600
C15A—H15C	0.9600	C15B—H15G	0.9600
C16A—H16A	0.9600	C16B—H16F	0.9600
C16A—H16B	0.9600	C16B—H16G	0.9600
C16A—H16C	0.9600	C16B—H16D	0.9600
			
C2A—C1A—C12A	110.6 (3)	C2B—C1B—C12B	109.8 (3)
C2A—C1A—C13A	111.7 (3)	C2B—C1B—C13B	110.6 (3)
C12A—C1A—C13A	106.3 (3)	C12B—C1B—C13B	106.6 (3)
C2A—C1A—H1A	109.4	C2B—C1B—H1AA	109.9
C12A—C1A—H1A	109.4	C12B—C1B—H1AA	109.9
C13A—C1A—H1A	109.4	C13B—C1B—H1AA	109.9
C7A—C2A—C3A	117.5 (3)	C3B—C2B—C7B	118.2 (3)
C7A—C2A—C1A	121.0 (3)	C3B—C2B—C1B	120.9 (3)
C3A—C2A—C1A	121.5 (3)	C7B—C2B—C1B	120.9 (3)
C4A—C3A—C2A	120.2 (3)	C4B—C3B—C2B	120.7 (4)
C4A—C3A—H3A	119.9	C4B—C3B—H3AA	119.7
C2A—C3A—H3A	119.9	C2B—C3B—H3AA	119.7
C3A—C4A—C5A	121.9 (4)	C3B—C4B—C5B	121.5 (3)
C3A—C4A—Br1A	119.5 (3)	C3B—C4B—Br1B	119.5 (3)
C5A—C4A—Br1A	118.5 (3)	C5B—C4B—Br1B	119.0 (3)
C4A—C5A—C6A	119.5 (4)	C6B—C5B—C4B	117.8 (3)
C4A—C5A—H5A	120.2	C6B—C5B—H5AA	121.1
C6A—C5A—H5A	120.2	C4B—C5B—H5AA	121.1
C7A—C6A—C5A	118.2 (4)	C5B—C6B—C7B	121.2 (4)
C7A—C6A—H6A	120.9	C5B—C6B—H6AA	119.4
C5A—C6A—H6A	120.9	C7B—C6B—H6AA	119.4
C6A—C7A—C2A	122.6 (4)	O8B—C7B—C6B	117.0 (3)
C6A—C7A—O8A	116.1 (3)	O8B—C7B—C2B	122.4 (3)
C2A—C7A—O8A	121.3 (3)	C6B—C7B—C2B	120.6 (3)
C7A—O8A—C9A	116.4 (3)	C7B—O8B—C9B	116.0 (3)
O8A—C9A—N10A	108.0 (3)	O8B—C9B—N10B	110.1 (3)
O8A—C9A—C16A	105.3 (3)	O8B—C9B—C16B	104.4 (3)
N10A—C9A—C16A	108.0 (3)	N10B—C9B—C16B	108.0 (3)
O8A—C9A—C13A	110.2 (3)	O8B—C9B—C13B	110.8 (3)
N10A—C9A—C13A	108.6 (3)	N10B—C9B—C13B	107.2 (3)
C16A—C9A—C13A	116.5 (3)	C16B—C9B—C13B	116.2 (3)
C11A—N10A—C9A	128.0 (3)	C11B—N10B—C9B	129.0 (3)
C11A—N10A—H10A	116.0	C11B—N10B—H10B	115.5
C9A—N10A—H10A	116.0	C9B—N10B—H10B	115.5
N10A—C11A—C12A	117.6 (3)	N10B—C11B—C12B	117.2 (3)
N10A—C11A—S1A	121.0 (3)	N10B—C11B—S1B	121.3 (3)
C12A—C11A—S1A	121.4 (3)	C12B—C11B—S1B	121.5 (3)
C11A—C12A—C1A	112.1 (3)	C11B—C12B—C1B	112.9 (3)
C11A—C12A—H12A	109.2	C11B—C12B—H12C	109.0
C1A—C12A—H12A	109.2	C1B—C12B—H12C	109.0
C11A—C12A—H12B	109.2	C11B—C12B—H12D	109.0
C1A—C12A—H12B	109.2	C1B—C12B—H12D	109.0
H12A—C12A—H12B	107.9	H12C—C12B—H12D	107.8
C14A—C13A—C9A	114.5 (3)	C9B—C13B—C1B	107.0 (3)
C14A—C13A—C1A	111.9 (3)	C9B—C13B—C14B	112.9 (3)
C9A—C13A—C1A	105.8 (3)	C1B—C13B—C14B	113.2 (3)
C14A—C13A—H13A	108.1	C9B—C13B—H13B	107.9
C9A—C13A—H13A	108.1	C1B—C13B—H13B	107.9
C1A—C13A—H13A	108.1	C14B—C13B—H13B	107.9
O14A—C14A—C15A	120.4 (4)	O14B—C14B—C15B	121.6 (4)
O14A—C14A—C13A	122.8 (3)	O14B—C14B—C13B	120.4 (3)
C15A—C14A—C13A	116.8 (3)	C15B—C14B—C13B	118.0 (3)
C14A—C15A—H15A	109.5	C14B—C15B—H15D	109.5
C14A—C15A—H15B	109.5	C14B—C15B—H15F	109.5
H15A—C15A—H15B	109.5	H15D—C15B—H15F	109.5
C14A—C15A—H15C	109.5	C14B—C15B—H15G	109.5
H15A—C15A—H15C	109.5	H15D—C15B—H15G	109.5
H15B—C15A—H15C	109.5	H15F—C15B—H15G	109.5
C9A—C16A—H16A	109.5	C9B—C16B—H16F	109.5
C9A—C16A—H16B	109.5	C9B—C16B—H16G	109.5
H16A—C16A—H16B	109.5	H16F—C16B—H16G	109.5
C9A—C16A—H16C	109.5	C9B—C16B—H16D	109.5
H16A—C16A—H16C	109.5	H16F—C16B—H16D	109.5
H16B—C16A—H16C	109.5	H16G—C16B—H16D	109.5
			
C12A—C1A—C2A—C7A	−98.7 (4)	C12B—C1B—C2B—C3B	−79.3 (4)
C13A—C1A—C2A—C7A	19.4 (4)	C13B—C1B—C2B—C3B	163.4 (3)
C12A—C1A—C2A—C3A	78.7 (4)	C12B—C1B—C2B—C7B	98.9 (4)
C13A—C1A—C2A—C3A	−163.2 (3)	C13B—C1B—C2B—C7B	−18.5 (4)
C7A—C2A—C3A—C4A	−1.6 (5)	C7B—C2B—C3B—C4B	1.5 (5)
C1A—C2A—C3A—C4A	−179.1 (3)	C1B—C2B—C3B—C4B	179.7 (3)
C2A—C3A—C4A—C5A	0.4 (6)	C2B—C3B—C4B—C5B	−0.1 (5)
C2A—C3A—C4A—Br1A	177.7 (3)	C2B—C3B—C4B—Br1B	−176.8 (3)
C3A—C4A—C5A—C6A	−0.3 (6)	C3B—C4B—C5B—C6B	−0.2 (5)
Br1A—C4A—C5A—C6A	−177.6 (3)	Br1B—C4B—C5B—C6B	176.5 (3)
C4A—C5A—C6A—C7A	1.4 (6)	C4B—C5B—C6B—C7B	−0.9 (5)
C5A—C6A—C7A—C2A	−2.7 (6)	C5B—C6B—C7B—O8B	−179.2 (3)
C5A—C6A—C7A—O8A	179.5 (3)	C5B—C6B—C7B—C2B	2.3 (5)
C3A—C2A—C7A—C6A	2.7 (5)	C3B—C2B—C7B—O8B	179.1 (3)
C1A—C2A—C7A—C6A	−179.7 (3)	C1B—C2B—C7B—O8B	0.9 (5)
C3A—C2A—C7A—O8A	−179.6 (3)	C3B—C2B—C7B—C6B	−2.5 (5)
C1A—C2A—C7A—O8A	−2.1 (5)	C1B—C2B—C7B—C6B	179.3 (3)
C6A—C7A—O8A—C9A	−164.1 (3)	C6B—C7B—O8B—C9B	164.9 (3)
C2A—C7A—O8A—C9A	18.1 (4)	C2B—C7B—O8B—C9B	−16.6 (4)
C7A—O8A—C9A—N10A	67.6 (3)	C7B—O8B—C9B—N10B	−69.1 (3)
C7A—O8A—C9A—C16A	−177.2 (3)	C7B—O8B—C9B—C16B	175.2 (3)
C7A—O8A—C9A—C13A	−50.9 (4)	C7B—O8B—C9B—C13B	49.4 (4)
O8A—C9A—N10A—C11A	−98.4 (4)	O8B—C9B—N10B—C11B	99.0 (4)
C16A—C9A—N10A—C11A	148.2 (3)	C16B—C9B—N10B—C11B	−147.6 (3)
C13A—C9A—N10A—C11A	21.1 (4)	C13B—C9B—N10B—C11B	−21.6 (4)
C9A—N10A—C11A—C12A	−4.2 (5)	C9B—N10B—C11B—C12B	1.5 (5)
C9A—N10A—C11A—S1A	175.8 (3)	C9B—N10B—C11B—S1B	−179.0 (3)
N10A—C11A—C12A—C1A	21.9 (4)	N10B—C11B—C12B—C1B	−16.3 (4)
S1A—C11A—C12A—C1A	−158.0 (3)	S1B—C11B—C12B—C1B	164.1 (2)
C2A—C1A—C12A—C11A	66.0 (4)	C2B—C1B—C12B—C11B	−69.2 (4)
C13A—C1A—C12A—C11A	−55.4 (4)	C13B—C1B—C12B—C11B	50.6 (4)
O8A—C9A—C13A—C14A	−58.5 (4)	O8B—C9B—C13B—C1B	−65.1 (4)
N10A—C9A—C13A—C14A	−176.6 (3)	N10B—C9B—C13B—C1B	55.1 (3)
C16A—C9A—C13A—C14A	61.2 (4)	C16B—C9B—C13B—C1B	176.0 (3)
O8A—C9A—C13A—C1A	65.2 (3)	O8B—C9B—C13B—C14B	60.1 (4)
N10A—C9A—C13A—C1A	−52.9 (3)	N10B—C9B—C13B—C14B	−179.8 (3)
C16A—C9A—C13A—C1A	−175.1 (3)	C16B—C9B—C13B—C14B	−58.9 (4)
C2A—C1A—C13A—C14A	76.9 (3)	C2B—C1B—C13B—C9B	47.7 (4)
C12A—C1A—C13A—C14A	−162.5 (3)	C12B—C1B—C13B—C9B	−71.6 (3)
C2A—C1A—C13A—C9A	−48.5 (4)	C2B—C1B—C13B—C14B	−77.2 (4)
C12A—C1A—C13A—C9A	72.2 (3)	C12B—C1B—C13B—C14B	163.4 (3)
C9A—C13A—C14A—O14A	92.4 (4)	C9B—C13B—C14B—O14B	−89.8 (4)
C1A—C13A—C14A—O14A	−28.0 (5)	C1B—C13B—C14B—O14B	31.9 (5)
C9A—C13A—C14A—C15A	−89.3 (4)	C9B—C13B—C14B—C15B	91.9 (4)
C1A—C13A—C14A—C15A	150.3 (3)	C1B—C13B—C14B—C15B	−146.4 (4)

### Hydrogen-bond geometry (Å, °)

**Table:**

*D*—H···*A*	*D*—H	H···*A*	*D*···*A*	*D*—H···*A*
N10A—H10A···O14B^i^	0.86	2.20	2.981 (4)	150
N10B—H10B···O14A^ii^	0.86	2.15	2.960 (4)	157

Symmetry codes: (i) *x*, *y*−1, *z*; (ii) *x*−1, *y*, *z*.

## References

[bb1] Biala, J., Czarnocki, Z. & Maurin, J. K. (2002). *Tetrahedron Asymmetry*, **13**, 1021–1023.

[bb2] Bilokin, Y. V., Kovalenko, S. N. & Chernykh, V. P. (1988). *Heterocycl. Commun.* **4**, 169–170.

[bb3] Konovalova, I. S., Zaremba, O. V., Kovalenko, S. S., Chernykh, V. P., Kovalenko, S. M., Baumer, V. N. & Shishkin, O. V. (2007). *Acta Cryst.* E**63**, o4906.

[bb4] Kovalenko, V. N. & Victorova, A. P. (2005). *Compendium of Medicinal Preparation*, p. 1920. Kiev: Morion.

[bb5] O’Callaghan, C. N., McMurry, T. B. H., O’Brien, J. E. & Draper, S. M. (1997). *J. Chem. Res.* **312**, 2101–2122.

[bb6] Raev, L. D., Frey, W. & Ivanov, I. C. (2004). *Synlett*, pp. 1584–88.

[bb7] Sheldrick, G. M. (2008). *Acta Cryst.* A**64**, 112–122. 10.1107/S0108767307043930 18156677

[bb8] Siemens (1991). *P3*, *XDISK* and *XPREP* Siemens Analytical X-ray Instruments Inc., Karlsruhe, Germany.

[bb9] Zaragoza Dorwald, F. (2000). US Patent 6136984 24 10.

[bb10] Zefirov, Yu. V. & Zorky, P. M. (1995). *Usp. Khim.* **64**, 446–460.

